# Constipation in Babies

**Published:** 1898-10

**Authors:** 


CONSTIPATION IN BABIES may be relieved by light massage of the abdomen, by a circular motion around the umbilicus, pressure being light, and exerted especially in the right iliac region. Each manipulation should last but ten minutes and should be done in the morning. The palm of the hand should be well oiled. For babies after the first year massage may be made with the finger tips over the large intestine from right to left.—*Monthly Retrospect of Medicine and Pharmacy.*

